# Ergebnisse einer Pilotstudie zur Rolle der Therapieerwartung bei der interdisziplinären multimodalen Schmerztherapie bei chronischem Rückenschmerz

**DOI:** 10.1007/s00482-021-00590-1

**Published:** 2021-10-07

**Authors:** Dustin Maser, Daniel Müller, Ulrike Bingel, Diana Müßgens

**Affiliations:** Klinik für Neurologie, Zentrum für Translationale Neuro- und Verhaltenswissenschaften und Zentrum für universitäre Schmerzmedizin, Universitätsklinikum Essen (AöR), Hufelandstraße 55, 45147 Essen, Deutschland

**Keywords:** Chronischer Rückenschmerz, Interdisziplinäre multimodale Schmerztherapie (IMST), Therapieerwartung, Placebo, Longitudinale Beobachtungsstudie, Chronic back pain, Interdisciplinary multimodal pain therapy (IMPT), Treatment expectancy, Placebo, Longitudinal observational study

## Abstract

**Hintergrund:**

Chronische Rückenschmerzen sind eine schwerwiegende und global sehr häufig auftretende Erkrankung mit enormen persönlichen sowie sozioökonomischen Auswirkungen. Die interdisziplinäre multimodale Schmerztherapie (IMST) ist eines der wenigen evidenzbasierten Behandlungsverfahren für chronische Schmerzen. Obwohl bekannt ist, dass Schmerzen sowie deren Chronifizierung und Behandlung von den persönlichen Erwartungen der Patienten beeinflusst werden, gibt es wenige etablierte Interventionen oder Richtlinien für eine aktive Modulation dieses Effekts.

**Ziel der Arbeit:**

Wir möchten mit dieser Arbeit die Rolle der Erwartung als Prädiktor für Schmerzen sowie schmerzbezogene Beeinträchtigung in der klinischen Praxis verdeutlichen und präsentieren hierzu beispielhaft explorative Pilotdaten einer Beobachtungskohorte unserer Klinik.

**Material und Methoden:**

Die Untersuchung zeigt erste Daten einer prospektiven longitudinalen Beobachtungsstudie bestehend aus bis zu 41 Patienten mit chronischen Rückenschmerzen, die im Setting einer IMST am Essener Rückenschmerz-Zentrum behandelt wurden. Es wurden Daten zum Zeitpunkt der Aufnahme (T0) und der Entlassung (T1) sowie drei Monate nach Therapieende (T2) erhoben. Primäre Endpunkte waren die Schmerzintensität und die Schmerzbeeinträchtigung. Zusätzlich erfassten wir die Therapieerwartung zum Zeitpunkt der Aufnahme als möglichen Prädiktor. Die Bedeutung der vor der Therapie erhobenen Therapieerwartung wurde mittels linearer Regression erfasst.

**Ergebnisse:**

Die IMST führte zu einer signifikanten Besserung in Bezug auf die Schmerzintensität und -beeinträchtigung. Der Effekt auf die Schmerzintensität war über den Zeitraum von drei Monaten nach Therapieende anhaltend und die Beeinträchtigung sank in diesem Zeitraum weiter signifikant.

**Diskussion:**

Erwartung war ein signifikanter Prädiktor für die Abnahme der Schmerzintensität und erklärte ca. 15 % der Varianz. In der klinischen Praxis sollten daher valide Methoden etabliert werden, negative Erwartungen zu reduzieren und positive Erwartungen zu fördern.

## Abstract (en)

**Background:**

Chronic low back pain is a serious persistent illness with profound personal and socioeconomic impact. Interdisciplinary multimodal pain therapy (IMPT) is one of the few evidence-based treatment options for chronic pain. Although it is known that pain perception, as well as its chronification and treatment are affected by patient expectations, only few clinical interventions or guidelines on how to modulate these effects exist.

**Objectives:**

The aim of this study was to demonstrate the impact of expectancy as a predictor for pain and related outcomes. To this end, we will present explorative pilot data from an observational cohort at our clinic.

**Methods:**

The study shows preliminary data of a prospective longitudinal observational study of up to 41 chronic back pain patients who followed an IMPT at the back pain center in Essen. Data were collected at admission (T0), at discharge (T1), and 3 months after discharge (T2). Primary outcomes were pain intensity and disability. Additionally, we measured treatment expectancy at admission. We used linear regression to analyze the impact of pretreatment expectancy on the primary outcome measures.

**Results:**

IMPT led to a significant improvement in pain intensity and disability. The effect on pain intensity was stable over three months after discharge and disability declined even further. Expectancy was a significant predictor of improvement in pain intensity and explained approximately 15% of the variance.

**Discussion:**

Expectancy is an important predictor of treatment outcome in IMPT. In clinical practice, valid methods should therefore be established to reduce negative and promote positive expectations.

## Hintergrund und Fragestellung

### Chronische Rückenschmerzen

Rückenschmerz gilt weltweit als eine der Hauptursachen für Aktivitätseinschränkungen und Arbeitsausfälle [26], was zu einer hohen sozioökonomischen und individuellen Belastung führt [17]. Etwa 90 % aller Rückenschmerzen sind unspezifisch und somit nicht zurückführbar auf einen einfachen pathoanatomischen Zusammenhang [39]. Persistieren Rückenschmerzen länger als drei bis sechs Monate gelten sie als chronisch [62]. Obwohl lediglich ca. 10–15 % aller Fälle chronifizieren [67], scheinen gerade diese Fälle hochgradig therapieresistent zu sein [21].

### IMST

Die interdisziplinäre multimodale Schmerztherapie (IMST) ist ein evidenzbasiertes Therapieverfahren bei chronischen Schmerzen [52] und gilt in Deutschland als Goldstandard zur Behandlung chronischer Schmerzen [27]. Viele nationale und internationale Studien bestätigen die Effektivität der IMST für unterschiedliche chronische Schmerzsyndrome [20, 23, 29, 57]. Dennoch zeigen einige Übersichtsartikel nur eine schwache oder moderate Evidenz und unterstreichen die Notwendigkeit qualitativ hochwertigerer Studien [16, 29, 45]. So gibt es im internationalen Raum immer noch keine einheitliche Definition für IMST, und evaluierte Therapien unterscheiden sich dadurch oft in Inhalt und Qualität [28]. Darüber hinaus ist unklar welche, der vielen Inhalte wirksam sind und wie sie mit Patientenvariablen interagieren. Erste Schritte wurden in Deutschland getätigt, indem detaillierte Konzepte für Struktur‑, Prozess- und Ergebnisparameter erarbeitet wurden [1, 2, 53]. Im internationalen Vergleich bietet die deutsche Schmerztherapielandschaft somit einen besonderen und chancenreichen Kontext, um die Effekte von Patientenvariablen unter vergleichsweise standardisierten Bedingungen zu untersuchen und diesbezügliche Interventionen zu entwickeln. Insbesondere die Therapierwartung scheint hier eine wichtige Rolle zu spielen.

### Die Rolle der Erwartung bei Schmerzen

Eine zunehmende Zahl internationaler Studien unterstreicht den Einfluss von Erwartung auf den Schmerz und seine Behandlung [24, 42, 49]. Die Erwartung gilt als eines der Kernelemente des Placebo- sowie Noceboeffekts [3] und kann somit einen positiven oder negativen Einfluss auf die Behandlung und den Krankheitsverlauf haben. Dies gilt in Anwesenheit eines bekannten Wirkfaktors, beispielsweise eines Medikaments, aber auch in seiner Abwesenheit. Therapieerwartungen können sowohl Schmerzen als auch die Wirkung einer analgetischen Behandlung verstärken oder abschwächen [8]. Dies lässt sich durch subjektive Patientenaussagen und durch Messungen der neuronalen Aktivität in den schmerzbezogenen Kortizes belegen [31]. Für Details siehe Asan et al. in diesem Themenheft.

Präoperative Erwartungen beeinflussen schmerzbezogene Ergebnisse bei unterschiedlichen chirurgischen Eingriffen [4], und sogar Scheinoperationen können zu einer Symptomlinderung führen [41, 44]. Erwartungen beeinflussen ebenfalls die Rehabilitation nach akuten Körpertraumata [46]. Positive Erwartungen gelten als protektiver Faktor im Übergang von akuten zu chronischen Schmerzen [42]. Auch bei chronischen Schmerzen ist der modulierende Einfluss von positiven Erwartungen belegt [14, 22, 70]. So unterstützen Erwartungen die Ergebnisse von Akupunktur [70], kognitiver Verhaltenstherapie [22] und multimodaler Schmerztherapie bei chronischen Schmerzen [14].

Auch negative Erwartungen beeinflussen das Therapieergebnis und sind ein häufiges Phänomen im klinischen Alltag [47]. So entwickeln Patienten nach entsprechender Aufklärung auch Nebenwirkungen, die nicht zum bekannten Nebenwirkungsspektrum dieses Medikaments gehören [8, 19]. Aversive Reize können ebenfalls verstärkt werden. Der Hinweis, ein Nadelstich könnte schmerzen, führt so zu einer Hyperalgesie [65]. Gleichsam können negative Erwartungen, Sorgen und Ängste bezüglich einer Behandlung unerwünschte Nebenwirkungen von medikamentösen Behandlungen verstärken [36, 38]. Die Erwartungshaltung ist also ein bedeutsamer Prädiktor für die Schmerztherapie, da sie das Therapieergebnis sowohl positiv als auch negativ beeinflussen kann.

### Ziele der Pilotstudie

Ziel dieser Pilotstudie war es, den Einfluss der Therapieerwartung auf das Ergebnis einer interdisziplinären multimodalen Schmerztherapie bei chronischen Rückenschmerzen zu untersuchen. Die Ergebnisse zielen darauf ab, auf das Ausmaß dieser Einflussgröße hinzuweisen. Darüber hinaus könnten sie eine Grundlage bilden, um Therapieergebnisse durch gezielte erwartungsmodulierende Interventionen zu optimieren.

## Studiendesign und Untersuchungsmethoden

### Behandlung am Essener Rückenschmerz-Zentrum

Das Essener Rückenschmerz-Zentrum existiert seit 2015 und behandelt regelhaft 130 Patienten jährlich mit der IMST. Patienten werden zumeist regional aus der Rhein-Ruhr-Region oder seltener auch überregional zu uns verwiesen.

Die IMST am Essener Rückenschmerz-Zentrum entspricht den Mindestvorgaben des amtlichen Operationen- und Prozedurenschlüssels (OPS) [6] und orientiert sich zusätzlich an den Empfehlungen zu Struktur- und Prozessparametern der Ad-hoc-Kommission „Interdisziplinäre Multimodale Schmerztherapie“ der Deutschen Schmerzgesellschaft e. V. [51]. Über die zwei geforderten Fachdisziplinen hinaus besteht das Behandlungsteam am Essener Rückenschmerz-Zentrum aus Medizinern unterschiedlicher Fachrichtungen, Physiotherapeuten und Psychotherapeuten, welche im stetigen interdisziplinären Austausch mit dem Patienten arbeiten. Die Behandlungstage zählen 14-21 Tage für stationäre und 10–15 Tage für teilstationäre Patienten und übersteigen damit die 7‑Tage Vorgabe des OPS. Die Anwendungen beinhalten unter anderem medikamentöse Einstellung, Psychotherapie, Physiotherapie, Psycho‑, Physio- sowie medizinische Edukation und Trainingstherapie. Auch dies überschreitet die Mindestanforderung von drei übenden Verfahren. Die Therapie wird vorrangig im Gruppensetting durchgeführt, wobei täglich mehrere Gruppen stattfinden (je 60 bis 90 min). Darüber hinaus erhält jeder Patient eine wöchentliche physiotherapeutische Einzelsitzung (je 30 min) sowie psychotherapeutische (je 45 min) und ärztliche Einzelgespräche (je 30 min). Entspannungs- oder Achtsamkeitsübungen werden am Ende eines jeden Tages angeleitet (je 30 min). An den Wochenenden erhalten die Patienten psychotherapeutische Aufgaben und sollen die erlernten Bewältigungsstrategien in Eigenarbeit üben.

Die Patienten, die zur IMST erscheinen, leiden oftmals unter therapieresistenten chronischen Rückenschmerzen und sind in ihrer Arbeitsfähigkeit oder Lebensqualität stark eingeschränkt. Häufig berichten die Patienten von jahrelangen „Ärzte-Odysseen“, sowie unzähligen misslungenen unimodalen Therapieversuchen, die von ambulanter Medikation bis hin zu mehrfachen fehlgeschlagenen Wirbelsäulenoperationen reichen. Bei den meisten Patienten werden die Schmerzen durch eine Kombination verschiedener biopsychosozialer Faktoren aufrechterhalten, und sie erfüllen damit zumeist die Kriterien der Diagnose *F45.41 chronische Schmerzstörung mit somatischen und psychologischen Faktoren* des ICD-10 [5]. Darüber hinaus leiden diese Patienten oft unter somatischen und/oder psychischen Komorbiditäten sowie unter einem Medikamentenfehlgebrauch.

### Studiendesign

Es wurden Patienten mit chronischen Rückenschmerzen im Alter von 29 bis 83 Jahren untersucht, die zwischen Januar 2019 und Februar 2020 am Essener Rückenschmerz-Zentrum im Rahmen einer IMST behandelt wurden. Die Indikation zur IMST wurde nach den Vorgaben des amtlichen Operationen- und Prozedurenschlüssels (OPS) [6] sowie den Empfehlungen der Ad-hoc-Kommission „Multimodale interdisziplinäre Schmerztherapie“ [12], gestellt. Bei entsprechender Indikation wurden Patienten zu einer zwei- oder dreiwöchigen IMST eingeladen, die im stationären oder teilstationären Setting erfolgen konnte. Die Auswahl des Settings wurde vorrangig von den Versicherungskonditionen und, in geringerem Maße, auch von den medizinischen Komorbiditäten der Patienten bestimmt. Eine Therapiegruppe bestand im Regelfall aus 6–7 stationären und 1–2 teilstationären Patienten. Ob eine zwei- oder dreiwöchige IMST durchgeführt wurde, hing vorrangig von strukturellen Variablen wie der Länge der Warteliste ab. Die Therapieinhalte waren jedoch unabhängig von Dauer und Setting. Die Patienten füllten den angepassten deutschen Schmerzfragebogen [50] im Rahmen des standardisierten Therapie-Monitorings zu vier verschiedenen Messzeitpunkten aus: Zur Aufnahme (T0), zur Entlassung (T1), sowie drei (T2), sechs (T3) und zwölf Monate (T4) nach der Therapie. Für die vorliegende Pilotstudie wurden ausschließlich die ersten drei Messzeitpunkte (T0–T2) inkludiert, da zum Zeitpunkt der Datensichtung noch nicht ausreichende Datenmengen für die anderen Messzeitpunkte erschlossen wurden. Nur Patienten mit vollständigem Datensatz und ausgefülltem Erwartungsfragebogen wurden eingeschlossen.

Grundsätzlich wurden alle Datenerhebungen online über die verschlüsselte Plattform PainPool® (smart‑Q Softwaresysteme GmbH) erhoben. Patienten erhielten maximal einen Monat im Voraus einen Link zur Internetplattform. Falls Patienten keine E‑Mail-Adresse besaßen oder den Fragebogen nicht rechtzeitig ausgefüllt haben, wurden die Daten handschriftlich am Tag der Aufnahme, noch bevor die eigentliche Therapie begann, erhoben. Zur Entlassung wurden die Daten am Ende des letzten Therapietags mit Tablets erhoben. In Ausnahmefällen, wenn Patienten z. B. technisch nicht mit dem Tablet zurechtkamen, wurde die Messung handschriftlich erhoben. Die Daten der Eingangserhebung zählten dann als auswertbar, wenn sie frühestens einen Monat vor der Therapie und spätestens am Aufnahmetag ausgefüllt wurden. Die Daten der Entlass-Erhebung galten dann als auswertbar, wenn sie frühestens am letzten Therapietag und spätestens bis zwei Monate nach der Therapie ausgefüllt wurden. T2 durfte frühestens nach zwei Monaten und bis spätestens fünf Monate nach Therapie erhoben werden.

Primäre Endpunkte der Studie waren Schmerzintensität sowie schmerzbedingte Beeinträchtigung. Zusätzlich erfassten wir die Erwartung zum Zeitpunkt der Aufnahme als Prädiktor. Die primäre Fragestellung war, ob die Erwartung vor Therapiebeginn Einfluss auf den Erfolg der IMST hat. Hierdurch ergaben sich folgende Hypothesen: (1) Schmerzintensität und -beeinträchtigung sinken im Verlauf der Messzeitpunkte. (2) Das Ausmaß dieser Symptomlinderung ist abhängig von der Erwartung der Patienten vor Therapiebeginn.

### Patienten

Im Untersuchungszeitraum vom 19.01.2019 bis zum 19.02.2020 haben 88 Patienten die Fragebogenbatterie mit dem Erwartungsfragebogen zu T0 ausgefüllt. Hiervon haben 16 den Fragebogen zu T1 nicht ausgefüllt. Zu T2 haben 31 weitere Patienten den Beeinträchtigungsfragebogen nicht ausgefüllt, und vier zusätzliche Patienten haben den Schmerzfragebogen zu T2 nicht beendet. Somit haben wir 37 vollständige schmerzbezogene und 41 vollständige beeinträchtigungsbezogene Datensätze gesammelt. Es ergibt sich ein Drop-out von 47 bzw. 51 (53,41 % bzw. 57,95 %). Zum Zeitpunkt T0 gibt es keine Unterschiede zwischen inkludierten Patienten und Drop-outs hinsichtlich der primären Endpunkte oder des Alters.

Tab. 1 zeigt die Verteilung der Patienten auf die primären Endpunkte hinsichtlich Alter, Arbeitsfähigkeit, Geschlecht, Wohnsituation, Medikamentenfehlgebrauch, primäre Endpunkte zu T0, Grad der Chronifizierung nach von Korff [68], Komorbiditäten, psychologischen Auffälligkeiten nach der Depression, Anxiety and Stress Scale (DASS) [48], Beeinträchtigung des habituellen Wohlbefindens (FW-7) [25], der gesundheitsbezogenen Lebensqualität (körperlich, geistig) nach der deutschen Version des Veterans RAND 12 (SF12) [30], Behandlungsdauer und -setting. Abgesehen von einem Patienten mit einem „failed back surgery syndrome“ (M96.1) trugen alle Patienten die Hauptdiagnose F45.41 – Chronische Schmerzstörung mit somatischen und psychischen Faktoren – ein. Unsere Stichprobe scheint hinsichtlich der erhobenen Variablen ungefähr der Normgruppe im „Handbuch Deutscher Schmerzfragebogen“ zu entsprechen [50]. Lediglich die geistige Gesundheit scheint etwas niedriger als in der dort genannten Normgruppe von Schmerzpatienten.

**Table 1:** 

	Schmerzintensität	Beeinträchtigung
***Alle Gruppen*** (*n* [%])	37 [100 %]	41 [100 %]
***Alter*** (M ± SD [min-max])	54,78 ± 15,20 [29–83]	55,46 ± 14,95 [29–83]
***Arbeitsunfähigkeit***^***a***^ (M ± SD [min-max])	47,32 ± 37,27 [0–92]	45,10 ± 36,14 [0–92]
***Geschlecht***		
**Weiblich **(*n* [%])	23 [62,2 %]	26 [63,4 %]
**Männlich **(*n* [%])	14 [37,8 %]	15 [36,6 %]
***Wohnsituation***		
**Alleine **(*n* [%])	9 [24,3 %]	10 [24,4 %]
**In Partnerschaft **(*n* [%])	22 [59,5 %]	24 [58,5 %]
**Anders **(*n* [%])	0 [0,0 %]	1 [2,4 %]
**NA** (*n* [%])	6 [16,2 %]	6 [14,6 %]
***Medikamentenfehlgebrauch***		
**Ja **(*n* [%])	9 [24,3 %]	9 [22,0 %]
**Nein **(*n* [%])	28 [75,7 %]	32 [78,0 %]
***Schmerzintensität T0*** (M ± SD [min-max])	66,49 ± 20,85 [20,00–83,3]	66,50 ± 20,12 [20,00–93,3]
***Beeinträchtigung T0*** (M ± SD [min-max])	70,45 ± 22,96 [13,33–100,00]	71,54 ± 22,01 [13,33–100,00]
***Grad der Chronifizierung (von Korff)***		
**1 **(*n* [%])	2 [5,4 %]	2 [4,9 %]
**2 **(*n* [%])	3 [8,1 %]	3 [7,3 %]
**3 **(*n* [%])	9 [24,3 %]	9 [22,0 %]
**4 **(*n* [%])	23 [62,2 %]	27 [65,9 %]
***Komorbiditäten***^***b***^ (M ± SD [min-max])	3,22 ± 2,25 [0–10]	3,39 ± 2,21 [0–10]
***Psychische Auffälligkeiten (DASS)***		
**Depression **(M ± SD [min-max])	6,92 ± 4,30 [1–16]	7,76 ± 4,92 [1–19]
*Klinisch auffällig* (*n* [%])	8 [21,6 %]	12 [70,7 %]
*Unauffällig* (*n* [%])	29 [78,4 %]	29 [29,3 %]
**Angst **(M ± SD [min-max])	4,32 ± 4,83 [0–19]	4,66 (± 4,76) [0–19]
*Klinisch auffällig* (*n* [%])	9 [24,3 %]	12 [70,7 %]
*Unauffällig* (*n* [%])	28 [75,7 %]	29 [29,3 %]
**Stress **(M ± SD [min-max])	7,92 ± 4,73 [1–19]	8,22 ± 4,86 [1–19]
*Klinisch auffällig* (*n* [%])	13 [35,1 %]	15 [63,4 %]
*Unauffällig* (*n* [%])	24 [64,9 %]	26 [36,6 %]
***Wohlbefinden (FW-7) ***(M ± SD [min-max])	15,19 ± 7,49 [2–32]	14,83 ± 8,33 [0–33]
**Beeinträchtigt **(*n* [%])	12 [32,4 %]	14 [34,1 %]
**Normal **(*n* [%])	25 [67,6 %]	27 [65,9 %]
***Lebensqualität (SF12)***		
**Körperl. Gesundheit **(M ± SD [min-max])	29,17 ± 9,89 [8,41–52,14]	29,13 ± 9,58 [8,41–52,14]
*1* *×* *SD niedriger*^*c*^ (*n* [%])^c^	8 [21,6 %]	9 [22,0 %]
*Norm Schmerzpatient*^*c*^ (*n* [%])	24 [64,9 %]	27 [65,9 %]
*1* *×* *SD höher* (*n* [%])	5 [13,5 %]	5 [12,2 %]
**Geist. Gesundheit**^c^ (M ± SD [min-max])	39,42 ± 13,35 [17,21–66,27]	38,33 ± 13,13 [17,21–66,27]
*1* *×* *SD niedriger*^*c*^ (*n* [%])	18 [48,6 %]	22 [53,7 %]
*Norm Schmerzpatient*^*c*^ (*n* [%])	12 [32,4 %]	12 [29,3 %]
*1* *×* *SD höher*^*c*^ (*n* [%])	7 [18,9 %]	7 [17,1 %]
***Setting***		
**Stationär **(*n* [%])	30 [81,1 %]	34 [82,9 %]
**Teilstationär **(*n* [%])	7 [18,9 %]	7 [17,1 %]
***Dauer***		
**2‑Wochen **(*n* [%])	9 [24,3 %]	11 [26,8 %]
**3‑Wochen **(*n* [%])	28 [75,7 %]	30 [73,2 %]

^a^Arbeitsunfähigkeit gemessen anhand Anzahl Tage, an denen in den letzten drei Monaten Arbeitsunfähigkeit vorlag

^b^Komorbiditäten wurden anhand der Diagnosen in den jeweiligen Arztbriefen erfasst und gezählt. Somatische Korrelate der Rückenschmerzen sowie Verdachtsdiagnosen wurden hierbei ausgeschlossen

^c^Bezieht sich auf die Validierungsstichprobe 2006, mit Mittelwerten und Standardabweichungen, aus dem Handbuch zum Deutschen Schmerzfragebogen [50]

### Fragebögen

Die Schmerzintensität sowie die schmerzbedingte Beeinträchtigung wurden mit dem Fragebogen zur Graduierung chronischer Schmerzen nach von Korff erhoben [68]. Hierbei wird ein zusammengefasster Schmerzwert als Mittelwert aus der aktuellen, maximalen und durchschnittlichen Schmerzbewertung über die letzten vier Wochen, auf einer elf Punkte umfassenden numerischen Ratingskala (NRS) [0–10, Anker: „kein Schmerz“–„unerträglicher Schmerz“], berechnet und mit 10 multipliziert. Für die schmerzbedingte Beeinträchtigung wird der Mittelwert aus der subjektiven Beeinträchtigung im Alltag, bei Freizeitaktivitäten und bei der Arbeit, bezogen auf die letzten drei Monate, auf einer elf Punkte umfassenden NRS [0–10, Anker: „keine Beeinträchtigung“-„völlige Beeinträchtigung“], berechnet und ebenfalls mit 10 multipliziert.

Der in dieser Studie untersuchte mögliche Prädiktor für den Therapierfolg ist die Therapieerwartung. Diese wurde mit einer ins Deutsche übersetzten und für das Setting angepassten Version des Credibility/Expectancies Questionnaire (CEQ) nach Devilly und Borkovec gemessen [15]. Der Erwartungswert setzt sich aus drei Werten zusammen. Die erste Frage bezieht sich auf die Gedanken des Patienten und erhebt auf einer elf Punkte umfassenden NRS [0–100 %] den Prozentsatz der Linderung, welchen die Patienten durch die Therapie erwarten. Die zweite Frage bezieht sich auf das Gefühl und fragt auf einer drei Punkte umfassenden Likert Skala [„überhaupt nicht“, „etwas“, „sehr“], wie sehr die Patienten wirklich fühlen, dass die Therapie helfen wird, ihre Symptome zu lindern. Die letzte Frage bezieht sich ebenfalls auf das Gefühl der Patienten und fragt auf einer elf Punkte umfassenden NRS [0–100 %] wie stark die Linderung, dem Gefühl der Patienten nach, tatsächlich nach der Therapie sein wird. Die Erwartungswerte werden in standardisierte Z‑Scores umgewandelt und als Summe verrechnet. Je höher der Z‑Score, desto größer ist auch die erwartete Linderung.

### Statistische Auswertung

Zunächst wurden Schmerzintensität und schmerzbedingte Beeinträchtigung sowie die Erwartungswerte mittels deskriptiver Statistik analysiert. Um die Vorannahmen der Normalverteilung zu überprüfen, wurden Shapiro-Wilk-Tests durchgeführt sowie QQ-Plots und Dichteplots beurteilt. Zur Überprüfung des Therapieeffekts wurden einfaktorielle ANOVAS mit Messwiederholungen für Schmerzintensität und Beeinträchtigung durchgeführt. Signifikante Veränderungen wurden anschließend durch Post-hoc-Paarvergleiche mit Bonferroni-Korrektur genauer definiert. Als Effektstärken wurden der generalisierte Eta-Quadrat für alle Messzeitpunkte sowie Cohens Delta für die Post-hoc-t-Tests bestimmt. Für die Prädiktorenanalyse wurden Änderungswerte für die primären Endpunkte berechnet. Hierzu wurde die für jeden Patienten die Steigung über alle drei Messzeitpunkte berechnet. Die resultierten Werte wurden mit −1 multipliziert. Somit gibt ein positiverer Wert eine größere Linderung an. Diese Linderungswerte wurden zunächst hinsichtlich ihrer Normalverteilung untersucht. Anschließend wurden lineare Regressionsanalysen mit jeweils der Schmerzlinderung sowie der Beeinträchtigungslinderung als abhängige und der Therapieerwartung als unabhängige Variable durchgeführt.

## Ergebnisse

### Deskriptive Statistik

Die Mittelwerte (M), Standardabweichungen (SD) sowie die Minima und Maxima der Schmerzintensität, der Beeinträchtigung und der Erwartung sind in Tab. 2 aufgeführt. Einstichproben-t-Tests zeigen, dass die Linderung der Schmerzintensität und die der Beeinträchtigung jeweils größer als 0 sind (Schmerzlinderung: t(36) = 5,3, *p* < 0,0001, d = 0,88, großer Effekt; Beeinträchtigungslinderung t(40) = 7,4, *p* < 0,0001, d = 1,15, großer Effekt). Da die Schmerzintensität sowie die Beeinträchtigung zu T0 und die Beeinträchtigungslinderung eine leichte Abweichung von der Normalverteilung zeigten, haben wir, wie von Tabachnick und Fidell [61] empfohlen, bei Linksschiefe eine invertierte Wurzeltransformation ($\sqrt{\left(\max \left(x\right)+1\right)-x}$) angewandt. Bei Rechtsschiefe haben wir eine einfache Wurzeltransformation benutzt. Da die Transformationen zu den gleichen signifikanten Effekten führen, rapportieren wir zugunsten der Interpretierbarkeit der Skalen die Analysen der nichttransformierten Werte.

**Table 2:** 

	Aufnahme (T0)		Entlassung (T1)		3‑Monate nach Entlassung (T2)		Linderung über alle Zeitpunkte	
	M ± SD	Range	M ± SD	Range	M ± SD	Range	M ± SD	Range
*Schmerzintensität*	66,5 ± 20,8	20,0–93,3	48,2 ± 16,4	10,0–76,7	43,2 ± 25,7	0,0–93,3	11,6 ± 13,6	-11,7–41,7
*Beeinträchtigung*	71,5 ± 22,1	13,3–100,0	60,2 ± 25,2	0,0–100,0	38,0 ± 25,9	0,0–100,0	16,8 ± 14,6	5,0–50,0
*Erwartung*^*a*^	0,5 ± 2,6	-5,0–5,2	–	–	–	–	–	–
*Erwartung*^*b*^	0,3 ± 2,6	-5,0–5,2	–	–	–	–	–	–

^a^ bezieht sich auf den Datensatz von 37 Patienten, welche die schmerzbezogenen Items der ersten drei Messzeitpunkte vollständig ausgefüllt haben

^b^ bezieht sich auf den Datensatz von 41 Patienten, welche die beeinträchtigungsbezogenen Items der ersten drei Messzeitpunkte vollständig ausgefüllt haben

### Verlauf der Schmerzintensität und der Schmerzbeeinträchtigung

**Figure 1:**
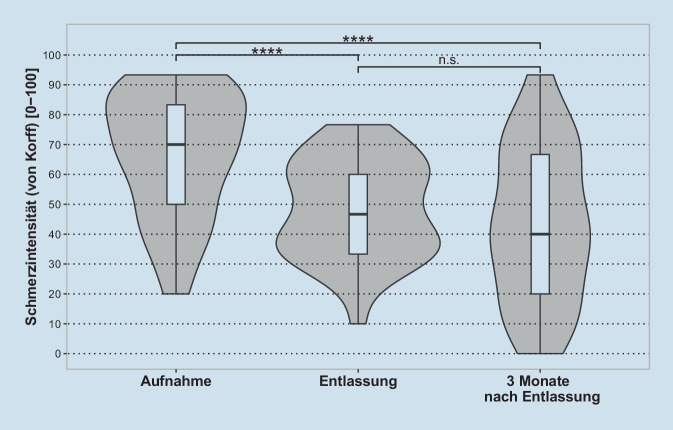

Eine einfache ANOVA mit Messwiederholungen zeigte einen signifikanten Haupteffekt von Messzeitpunkt auf die Schmerzintensität (F(1,39, 50,08) = 22,61, *p* < 0,0001, η^2^_g_ = 0,18, mittlerer Effekt) (Abb. 1). Post-hoc-t-Tests für abhängige Stichproben zeigten, dass die Schmerzintensität zwischen dem Messzeitpunkt der Aufnahme (M = 66,5, SD = 20,8) und der Entlassung (M = 48,2, SD = 16,4) (t(36) = 8,5, *p* < 0,0001, d = 1,39, großer Effekt) sowie zwischen der Aufnahme und drei Monate nach Entlassung (M = 43,2, SD = 25,7) (t(36) = 5,3, *p* < 0,0001, d = 0,88, großer Effekt) signifikant sank. Es gab keinen signifikanten Unterschied zwischen Entlassung und drei Monate nach Entlassung (t(36) = 1,2, *p* = 0,68).

**Figure 2:**
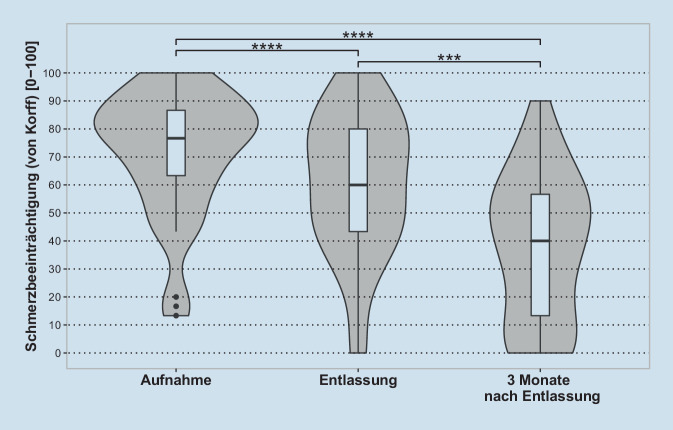

Analog zur Schmerzintensität zeigte eine weitere einfache ANOVA mit Messwiederholungen einen signifikanten Haupteffekt von Messzeitpunkt auf die schmerzbedingte Beeinträchtigung (F(1,24,49,64) = 36,43, *p* < 0,0001, η^2^_g_ = 0,25; mittlerer Effekt) (Abb. 2). Post-hoc-t-Tests für abhängige Stichproben zeigten, dass die Beeinträchtigung zwischen dem Messzeitpunkt der Aufnahme (M = 71,5, SD = 22,1) und der Entlassung (M = 60,2, SD = 25,2) (t(40) = 6,0, *p* < 0,0001, d = 0,94, großer Effekt), zwischen Entlassung (M = 60,2, SD = 25,2) und drei Monate nach Entlassung (M = 38,0, SD = 25,9) (t(40) = 4,6, *p* < 0,001,d = 0,71, mittlerer Effekt) sowie zwischen der Aufnahme (M = 71,5, SD = 22,1) und drei Monate nach Entlassung (M = 38,0, SD = 25,9) (t(40) = 7,4, *p* < 0,0001, d = 1,15, großer Effekt) signifikant sank.

### Zusammenhang zwischen Therapieerwartung und Schmerzlinderung

**Figure 3:**
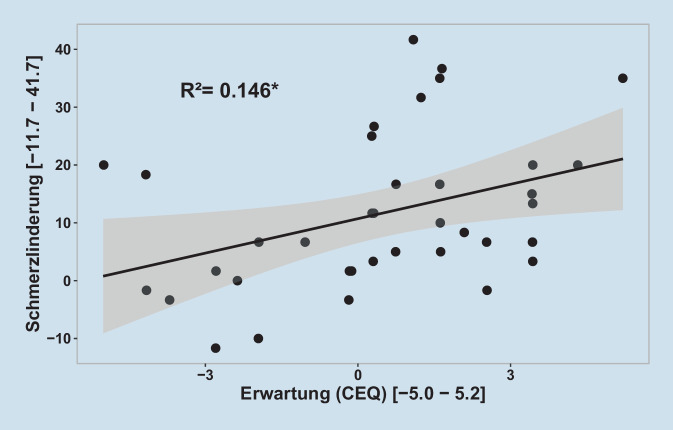

Die Regressionsanalyse zeigte einen signifikanten Zusammenhang zwischen der Therapieerwartung vor Beginn der Behandlung und der Schmerzlinderung über die drei Messzeitpunkte (β = 1,98, t(35) = 2,4, *p* < 0,05) (siehe Tab. 3). Die individuelle Therapieerwartung, erhoben mit der Erwartungsskala des CEQ, erklärte 14,6 % der Varianz in Schmerzlinderung (R^2^ = 0,15, F(1,35) = 6,00, *p* < 0,05, f^2^ = 0,18, mittlerer Effekt) (Abb. 3). Es zeigte sich kein signifikanter Zusammenhang zwischen der Therapieerwartung und der Beeinträchtigungslinderung.

**Table 3:** 

	Einfluss auf Schmerzlinderung		Einfluss auf Beeinträchtigungslinderung	
	Koeffizient	SD	Koeffizient	SD
*Konstante*	10,72***	–	4,47***	–
*Erwartung*	1,98*	0,81	0,12 (n. s.)	0,10
*R*^*2*^	0,15	–	0,04	–
*Korr. R*^*2*^	0,12	–	0,01	–
*F(1,35)/(1,39)*	6,00*	–	1,56 (n. s.)	–

*p < 0,05, ***p < 0,001

## Diskussion

In diesem Artikel diskutierten wir kurz die bestehende Literatur zu Erwartungseffekten bei der Behandlung von Schmerzerkrankungen und erweiterten diese durch exemplarische Daten der IMST unseres Hauses. Dies stellt einen ersten Nachweis dieser Einflussgröße in der besonderen schmerztherapeutischen Strukturlandschaft in Deutschland dar.

### Effektivität der IMST

In Übereinstimmung mit vorherigen Studien deuten auch unsere Daten auf die Effektivität der IMST für chronischen Rückenschmerzpatienten hin. Die hier gemessenen Effektstärken übersteigen die eher kleinen Effektstärken, die international beschrieben wurden [29]. Möglicherweise begründet sich dies in den vergleichsweise günstigen Rahmenbedingungen der IMST in Deutschland. Die hier erarbeiteten Konzepte zu Struktur‑, Prozess- und Ergebnisparametern fehlen im internationalen Raum noch, was die dortige Standardisierung und Evaluation erschwert [53]. Diese Annahme wird durch ähnliche Ergebnisse vergleichbarer deutscher Beobachtungsstudien gestützt [9, 43, 54].

### Erwartung als Prädiktor für Schmerzlinderung

Der Einfluss von Erwartung auf die Behandlung von Schmerzen ist bekannt bei Medikamenten [8], Akupunktur [70], kognitiver Verhaltenstherapie [22] sowie multimodaler Schmerztherapie [14, 63] und deckt sich somit mit unseren Ergebnissen. Die hier beobachtete mittlere Effektstärke in Verbindung mit 15 % erklärter Varianz verdeutlicht, dass die Erwartungshaltung des Patienten einen nicht zu vernachlässigenden Teil des Behandlungserfolgs ausmacht. Da es sich jedoch bei der Pilotstudie um eine Beobachtungsstudie handelt, sind keine Aussagen über die Kausalität dieses Zusammenhangs möglich. Auch können wir nicht erschließen, wie die Varianz in der Erwartung unserer Patienten zustande kommt.

Erwartungen können u. a. durch Vorerfahrungen, Konditionierungsprozesse, verbale Suggestion und soziale Lernprozesse, sowohl explizit als auch implizit, entstehen [7, 13]. Es liegt nahe, dass interindividuelle Unterschiede in Patientendispositionen und Vorerfahrungen zu divergenten Erwartungsprofilen und damit zu variierenden Ergebnissen führen. Bräscher et al. (2018) vermuten, dass konditionierungsinduzierte Erwartungseffekte eine große Rolle bei chronischen Schmerzpatienten spielen [10]. Eigenschaften von Schmerzpatienten, wie defizitäre Extinktionsfähigkeit oder eine Historie fehlgeschlagener Behandlungsversuche, könnten Erwartungseffekte zum negativen Pol verschieben. Auch morphologische Unterschiede im Kortex von chronischen Schmerzpatienten [40] könnten die erwartungsinduzierte deszendierende Schmerzhemmung stören. Zudem zeigen unterschiedliche Subgruppen von Schmerzpatienten variierende psychosoziale Profile [11, 18, 64], welche wiederum divergente Erwartungstendenzen implizieren. So können ungünstige Überzeugungen eine Chronifizierung fördern [66] und, wenn sie nicht adressiert werden, die Behandlung stören [69].

Um den Einfluss von Erwartungen therapeutisch zu nutzen, wurden bereits erwartungsmodulierende Interventionen entwickelt. So haben z. B. Rief und Glombiewski (2016) einen erwartungsfokussierten Therapieansatz für die psychotherapeutische Praxis entwickelt [55]. Hierdurch können positive Erwartungen induziert und gefördert werden. In diesem Rahmen sind psychoedukative Gruppen, Erfolgsberichte von vorherigen Patienten (soziales Lernen) oder hypnotherapeutische Suggestionen denkbar. Andererseits könnten pathogene oder generalisierte negative Erwartungen expliziert und adressiert werden. Hierfür wären Methoden der kognitiven Umstrukturierung, aber auch Reizexpositionen denkbar [55]. Durch derartige Interventionen ließen sich bereits Erfolge bei chronischen Rückenschmerzen erzielen [32, 35, 37, 59, 71]. Zukünftige kontrollierte Interventionsstudien sollten untersuchen, welche Erwartungsmodulationen am effektivsten sind und welche interindividuellen Patientenvariablen hierbei berücksichtigt werden sollten. Für eine Übersicht möglicher Strategien zur Optimierung von Erwartungseffekten siehe auch Bingel et al. (2020) [7]. Die Modulierbarkeit der Erwartungen legt nahe, solche Interventionen zu validieren und ihr therapeutisches Potenzial gezielt zu nutzen.

### Erwartung hat keinen Einfluss auf Beeinträchtigung

Der fehlende Nachweis eines Zusammenhangs zwischen der Besserung der Beeinträchtigung und der Erwartung widerspricht scheinbar den Ergebnissen anderer Studien [14, 63]. Unsere Patienten schienen hinsichtlich der Beeinträchtigung, unabhängig von spezifischen Erwartungen, zu profitieren. Eine mögliche Erklärung ist die Beschaffenheit des CEQ, dessen Items das Ausmaß der erwarteten Linderung ermitteln. Möglichweise beziehen Patienten diese Formulierung vorrangig quantitativ auf die Schmerzintensität. Der Fragebogen wäre insofern nicht geeignet, allgemeine oder beeinträchtigungsbezogene Therapieerwartungen zu erfassen. Kube et al. (2017) weisen darauf hin, dass Erwartungen keinen allgemeinen additiven Effekt auslösen, sondern eher spezifische Erwartungen mit den eigentlichen Wirkfaktoren einer Behandlung interagieren [33]. Für zukünftige Studien empfehlen wir deshalb die Verwendung von Fragebögen, die unterschiedliche Erwartungen und Vorerfahrungen bzgl. unterschiedlicher Therapieelemente und -effekte getrennt voneinander erfassen. Ein Beispiel hierfür wäre die Generische Rating Scale für vorherige Behandlungserfahrungen, Erwartungen und Effekte (GEEE) [56].

### Limitationen

Bei dieser Pilotstudie handelt es sich um eine prospektive longitudinale Beobachtungsstudie. Da die Daten im klinischen Alltag erhoben und genutzt wurden, sind sie weder randomisiert noch kontrolliert. Angesichts der gewöhnlichen Persistenz von chronischen Rückenschmerzen [60] erscheint es jedoch unwahrscheinlich, dass die hier gefundenen Effekte lediglich den spontanen Krankheitsverlauf beschreiben. Die Verwendung des von-Korff-Fragebogens ist aus folgenden Gründen kritisch zu sehen: Die Schmerzintensität-Skala erfasst den Zeitraum der letzten vier Wochen, die Skala der Beeinträchtigung den Zeitraum der letzten drei Monate. Beide Zeiträume übersteigen somit den Zeitraum der Therapie. Dass die Ergebnisse dennoch deutliche Besserungen nach drei bzw. zwei Wochen Therapie zeigen, ist möglicherweise die Folge des subjektiven Eindrucks der Patienten, welcher sich nicht zwangsläufig an objektivierbaren Zeitabschnitten orientierte. Wir möchten zudem darauf hinweisen, dass die Besserungen bis drei Monate nach Therapieende zumindest persistierten und teilweise weiter zunahmen.

Darüber hinaus schien die geistige Gesundheit in unserer Stichprobe geringer zu sein als die von anderen Schmerzpatienten. Dies muss jedoch nicht bedeuten, dass die gefundenen Erwartungseffekte nur oder besonders bei Patienten mit reduzierter geistiger Gesundheit wirken. Da ähnliche Erwartungseffekte bei einer Vielzahl von somatischen Beschwerden bekannt sind, ist anzunehmen, dass es sich hierbei nicht vordergründig um subgruppenspezifische, sondern um allgemeingültige Phänomene handelt. Jedoch gibt es durchaus Hinweise, dass gewisse psychologische Eigenschaften und psychische Zustände die modulierende Wirkung von Erwartungen beeinflussen können [7]. So können Anspannung, Stress und negativer Affekt die Wirkung von positiver Erwartung hemmen und die von negativer Erwartung verstärken. Auch mehr persistierende Zustände wie Ängste und Depressivität zeigen ähnliche Auswirkungen [34, 58]. Wenn reduzierte geistige Gesundheit einen ähnlich hemmenden Einfluss wie Depressivität hat, dann könnte es sein, dass der Effekt von Erwartung auf das Ergebnis durch unsere Ergebnisse noch unterschätzt wird. Zukünftige Studien sollten die Übertragbarkeit unserer Ergebnisse auch auf andere Patientenkollektive und die mediierende Wirkung von positiver geistiger Gesundheit überprüfen.

Des Weiteren sei zu erwähnen, dass wir keine Aussage über den Verlauf und den Einfluss von Erwartung der Patienten treffen können, die nicht alle Messzeitpunkte eingehalten haben. Zumindest scheinen sie sich zum Einschlusszeitpunkt nicht signifikant hinsichtlich der primären Endpunkte unterschieden zu haben. Dies ist eine generelle Limitation klinischer Studien.

## Ausblick und Fazit für die Praxis

Die bereits metaanalytisch belegte, klinisch relevante Effektivität der IMST spiegelt sich auch in unserer untersuchten Kohorte wider. Unsere Pilotuntersuchung bestätigt einen bereits in anderen experimentellen und klinischen Kontexten dokumentierten Zusammenhang zwischen der Therapieerwartung und dem Therapieergebnis. Auch wenn unsere Untersuchung aus methodischen Gründen keinen kausalen Zusammenhang der genannten Faktoren herstellen kann, unterstützt unsere Beobachtung in Zusammenschau mit experimentellen und kontrollierten klinischen Untersuchungen die Bedeutung der Therapieerwartung als eine wichtige Einflussgröße des Behandlungserfolgs. Obwohl diese Einflussgröße bereits in einer Vielzahl von experimentellen Studien beschrieben und untersucht wurde, wird sie aktuell in der klinischen Praxis kaum systematisch genutzt. Die hier vorgestellte Untersuchung soll dazu motivieren, die Bedeutung der Erwartung im schmerztherapeutischen Kontext inklusive der IMST in größeren Kohorten besser und systematischer zu untersuchen. Hierbei sind sowohl die Mechanismen der Erwartungsbildung als auch deren Effekte auf den Therapieerfolg von Bedeutung. Eine weitere wesentliche Herausforderung ist das Verständnis von interindividuellen Unterschieden der Erwartung und von Erwartungseffekten. Ein besseres Verständnis dieser Einflussgrößen sowie von dynamischen Veränderungen der Erwartung im longitudinalen Verlauf von Behandlungen sind die Grundlage, um die individuelle Therapieerwartung gezielt und zum Wohle des Patienten zu modulieren. Solche gezielten Interventionen könnten ein fester Bestandteil in der Schmerztherapie, ganz besonders im Rahmen der „kognitiv-behavioralen“ Elemente der IMST, sein. In einer weiterführenden Studie planen wir, den Einfluss einer erwartungsmodulierenden Intervention auf den Effekt der IMST zu untersuchen. Hierbei wird eine Gruppe, welche die erwartungsmodulierende Intervention erhält, mit der gewohnten IMST-Gruppe verglichen. Zusätzlich planen wir hierbei die laufende Warteliste der IMST als Kontrollgruppe zu nutzen.
